# Ehrlichiosis Manifesting as Septic Shock and Respiratory Failure With Alveolar Hemorrhage in an Immunocompetent Patient

**DOI:** 10.7759/cureus.87581

**Published:** 2025-07-09

**Authors:** Katherine R Sommers, Ronald G Washburn

**Affiliations:** 1 Department of Internal Medicine, Wake Forest School of Medicine, Winston-Salem, USA; 2 Department of Infectious Diseases, Wake Forest School of Medicine, Winston-Salem, USA

**Keywords:** alveolar hemorrhage, doxycycline, ehrlichia chaffeensis, ehrlichiosis, lone star tick

## Abstract

Ehrlichiosis is a bacterial infection transmitted through the bite of an infected lone star tick. The disease presents as a flu-like illness that is typically mild and self-limited. However, it can progress to severe disease with multiorgan involvement and numerous complications. We present a case of a 75-year-old immunocompetent female patient who developed septic shock and respiratory failure with alveolar hemorrhage after tick exposure. She was diagnosed with ehrlichiosis based on molecular testing via polymerase chain reaction (PCR) and completed a 10-day course of doxycycline with clinical improvement. Alveolar hemorrhage is a rare but serious complication of ehrlichiosis. This case emphasizes the importance of detailed history taking and the value of pattern recognition to facilitate timely diagnosis and treatment of ehrlichiosis, which is necessary for improved patient outcomes.

## Introduction

*Ehrlichia chaffeensis* is an intracellular Gram-negative bacterium transmitted to humans by infected ticks, primarily the lone star tick, which is endemic to the southeastern and south-central regions of the United States. Ehrlichiosis typically manifests as a flu-like illness with nonspecific symptoms such as fever, headache, and myalgias. Laboratory findings may reveal pancytopenia, elevated liver function tests, and acute kidney injury [1]. Though many cases are self-limited, they can progress to severe infection with multiorgan involvement, including respiratory failure, septic shock, and hepatitis [1]. Clinicians must maintain heightened awareness of ehrlichiosis, especially in endemic regions, to facilitate prompt recognition, timely diagnosis, and treatment to improve patient outcomes and prevent complications.

## Case presentation

A 75-year-old female patient residing in North Carolina with a medical history significant for mitral valve prolapse, gastroesophageal reflux disease, and osteopenia presented with two weeks of flu-like symptoms, including lightheadedness, shortness of breath, myalgias, and night sweats. She reported no recent travel or known sick contacts. On presentation, she was hypoxic, requiring supplemental oxygen, and tachycardic. Physical examination revealed no rash.

Initial laboratory evaluation was notable for leukopenia, thrombocytopenia, and elevated liver transaminases (Table 1). Computed tomography (CT) imaging of the chest, abdomen, and head was unremarkable. She was admitted for further workup of hypoxia. Further investigation into the elevated liver enzymes revealed biliary sludge on the right upper quadrant ultrasound, normal creatinine kinase, and a negative hepatitis profile. Thrombocytopenia persisted despite normalization of white blood cell count. Further laboratory studies revealed inflammation, as evidenced by elevated ferritin and lactate dehydrogenase, which serve as nonspecific markers of systemic inflammation (Table 1).

**Table 1:** Laboratory testing on various days during hospitalization demonstrated persistent thrombocytopenia despite normalization of white blood cell count, improvement of liver function testing after initiation of treatment, and resolution of acute kidney injury by day of discharge.

Laboratory studies		Day 1	Day 8	Day 14	Day of discharge	Normal range
Complete blood count	White blood count	2.72	18.88	6.89	6.22	4.40 - 11.00 10*3/uL
	Hemoglobin	13.0	8.8	8.1	7.5	12.3 - 15.3 g/dL
	Platelet count	131	172	387	427	150 - 450 10*3/uL
Complete metabolic panel	Creatinine	1.17	2.38	3.68	1.40	0.60 - 1.20 mg/dL
	Aspartate aminotransferase	133	91	21	--	13 - 39 U/L
	Alanine aminotransferase	84	53	21	--	7 - 52 U/L
	Alkaline phosphatase	153	216	114	--	34 - 104 U/L
Additional testing	Ferritin	728	--	--	--	11 - 307 ng/mL
	Lactate dehydrogenase	489	--	--	--	140 - 271 U/L

On day 2 of hospitalization, she developed recurrent fevers, peaking at 104.0°F, which were complicated by new-onset atrial fibrillation with rapid ventricular response. At that time, empiric broad-spectrum antibiotics, including vancomycin, cefepime, and metronidazole, were started for sepsis of unclear origin. Despite antimicrobial therapy, she remained febrile and clinically deteriorated, developing septic shock requiring transfer to the intensive care unit (ICU) for vasopressor support. 

**Figure 1:**
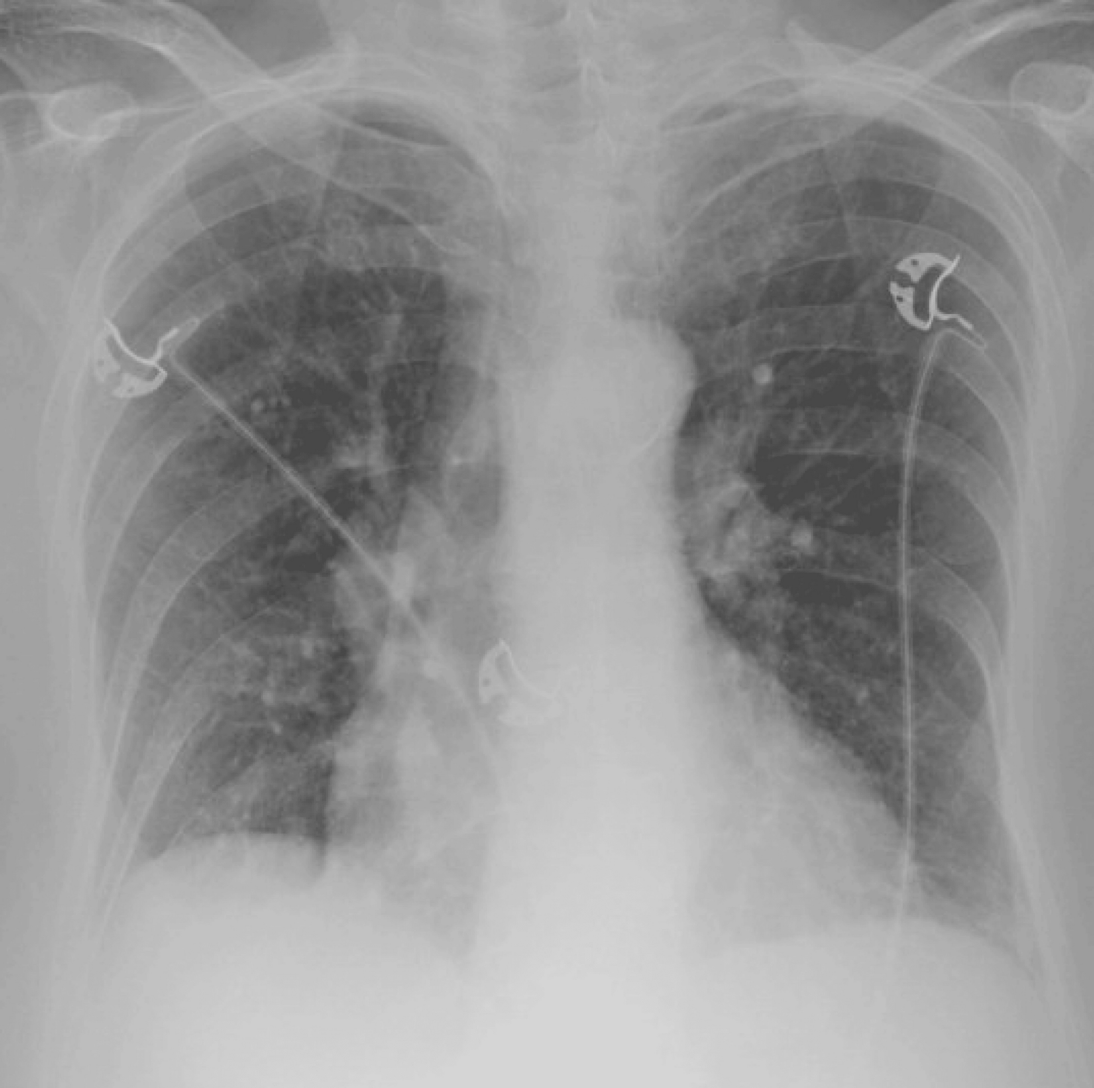
Chest radiograph on day 1 of hospitalization demonstrated mild bilateral infiltrates.

**Figure 2:**
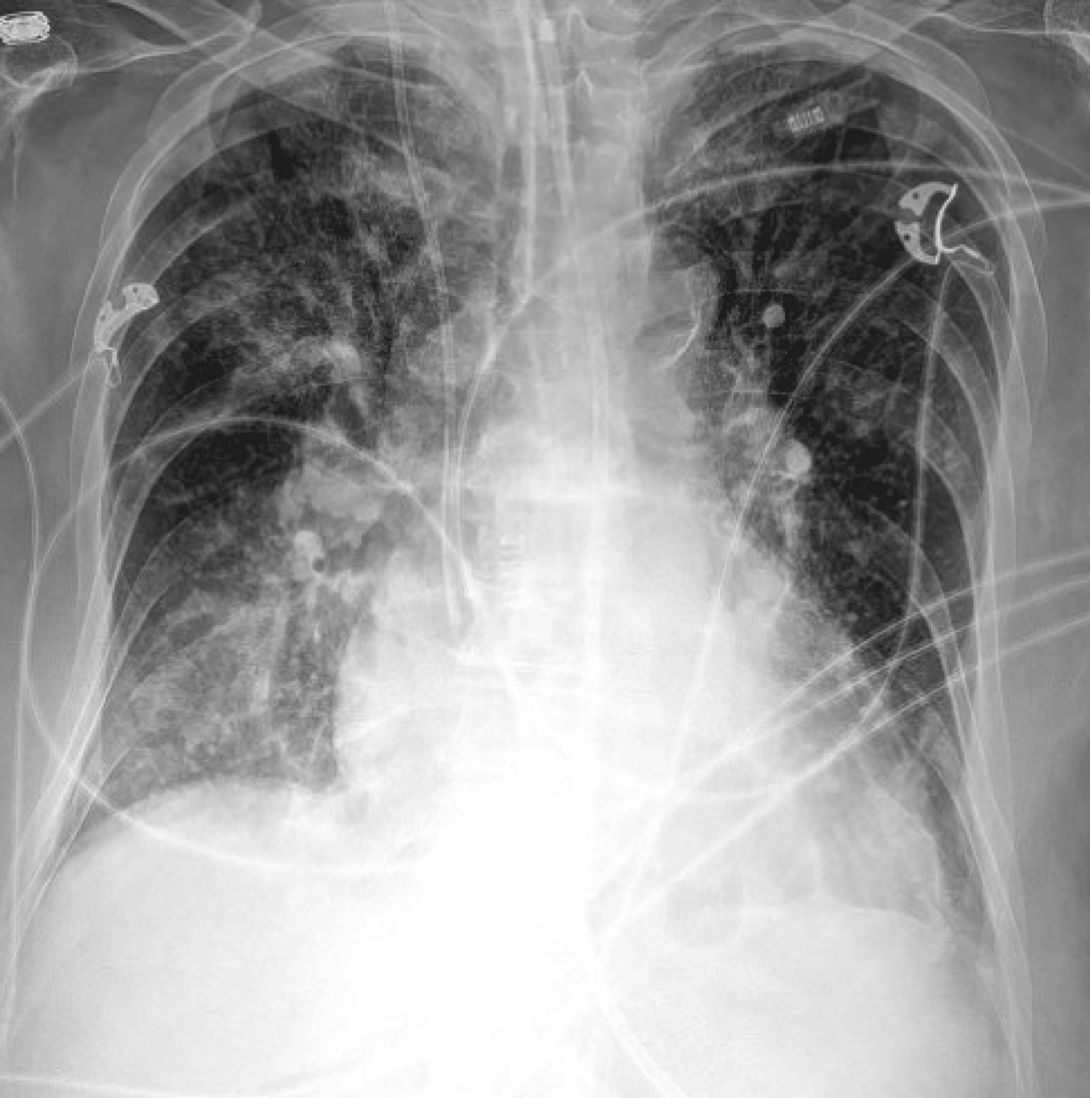
Chest radiograph on day 10 of hospitalization showed progression of bilateral infiltrates with worsening pulmonary edema.

The infectious diseases team was consulted, and further history from family members revealed the patient had recently removed a tick from her pet cat and had been exposed to mulch while gardening. Given these exposures and the severity of the patient’s condition, there was concern for disseminated fungal, tick-borne, or viral infection. Empiric amphotericin, doxycycline, and acyclovir were added to her antimicrobial regimen. The patient developed increasing oxygen requirements, requiring additional respiratory support that ultimately necessitated endotracheal intubation and mechanical ventilation. Serial chest radiographs demonstrated progressive bilateral infiltrates and pulmonary edema (Figures 1-2). Pulmonology was consulted, and a bronchoscopy was performed with findings consistent with diffuse alveolar hemorrhage (Table 2). The patient had fluctuating mental status, causing concern for central nervous system (CNS) infection. However, due to her critical condition, a lumbar puncture was not feasible. A comprehensive infectious workup was initiated and revealed negative testing for fungal or viral pathogens but returned positive for *Ehrlichia chaffeensis* by polymerase chain reaction (PCR) (Table 2).

**Table 2:** Laboratory investigations as part of the infectious workup, including serum and urine testing as well as bronchoscopy results, demonstrated Ehrlichia positivity with negative viral and fungal panels. EBV: Epstein-Barr virus; PCR: polymerase chain reaction

Laboratory studies		Result
Respiratory testing	Respiratory pathogen panel	Negative
Serum and urine testing	Blood culture	Negative
	EBV quantitative PCR	Negative
	Cytomegalovirus quantitative PCR	Negative
	Parvovirus B19 quantitative PCR	Negative
	*Toxoplasma gondii* PCR	Negative
	Blastomycosis serum and urine antigen	Negative
	Histoplasma galactomannan urine and serum antigen	Negative
	Aspergillus galactomannan antigen	Negative
	Cryptococcal antigen	Negative
	*Streptococcus pneumonia* urine antigen	Negative
	*Legionella pneumophilia* urine antigen	Negative
	*Leptospira *antibody IgM	Negative
	*Bartonella *antibody profile	Negative
	Lyme disease total antibody IgM/IgG	Negative
	Spotted fever group antibodies IgM/IgG	Negative
	*Ehrlichia chaffeensis* PCR	Positive
	*Anaplasma phagocytophilum* PCR	Negative
Bronchoscopy with bronchoalveolar lavage	Cell count	Bloody fluid
		111 nucleated cells
	Cell differential	Neutrophil 78%
		Lymphocyte 8%
		Monocyte 13%
		Basophil 1%
	Respiratory culture	Negative
	Acid-fast smear and culture	Negative
	Fungal culture	Negative
	Pneumonia panel PCR	Negative
	Cytology	No malignant cells
	Tissue exam	Few bland cells with neuroendocrine features

Following confirmation of the diagnosis, all antimicrobials were discontinued except doxycycline. The patient clinically improved, vasopressors were discontinued, and supplemental oxygen was quickly weaned off after self-extubating. She was subsequently transferred out of the ICU with continued improvement, including normalization of liver function testing and platelet count. Her hospital course was complicated by an acute kidney injury (Table 1). She completed a 10-day course of doxycycline prior to discharge and returned home with family to continue recovery given prolonged hospitalization and residual debility. By the day of discharge, acute kidney injury had completely resolved, and the patient was near her baseline functional status. 

## Discussion

Ehrlichiosis presents as a nonspecific, flu-like illness characterized by fevers, chills, fatigue, headache, and myalgias [2]. *Ehrlichia chaffeensis* spreads hematogenously after being transmitted through the bite of an infected tick, targeting the peripheral blood system monocytes and macrophages. This can result in widespread organ involvement, including the brain, lungs, and kidneys [3]. As demonstrated in our case, severe manifestations including respiratory failure, septic shock, and multiorgan involvement are possible, especially in individuals with advanced age or immunocompromising conditions [4]. Notably, there is a higher risk of severe disease and death in patients who experience a delay in treatment with doxycycline [5]. 

Ehrlichiosis is diagnosed based on clinical suspicion paired with laboratory confirmation. Diagnostic modalities include peripheral blood smear examination for intracytoplasmic inclusions, molecular testing via PCR, in vitro testing, and serologic antibody testing. Due to the risk of progression to severe disease or complications, doxycycline should be initiated as soon as the diagnosis is suspected while awaiting laboratory confirmation. [6]. Severe complications of ehrlichiosis can include acute respiratory distress syndrome (ARDS), septic shock, kidney failure, respiratory failure, or meningoencephalitis. Less commonly, patients may develop alveolar hemorrhage and vascular congestion, particularly in severe disease [7]. Treatment consists of seven to 10 days of doxycycline, with clinical improvement often demonstrated 24-48 hours after initiating therapy.

Our patient had classic clinical and laboratory manifestations of ehrlichiosis, including a flu-like prodrome, pancytopenia with persistent thrombocytopenia, and elevated liver function tests. She had relevant exposure to ticks via a pet, resided in an endemic region, and her presentation coincided with a high-incidence season. She rapidly developed severe disease, including acute hypoxic respiratory failure requiring mechanical ventilation. Bronchoscopy revealed diffuse alveolar hemorrhage, an infrequently described complication of ehrlichiosis, especially in an immunocompetent patient. Tissue pathology obtained during bronchoscopy demonstrated neuroendocrine cells, an incidental finding that pulmonology consultants felt could be in the setting of lung inflammation and recommended following up in the outpatient setting for repeat imaging. 

More common pulmonary manifestations include bilateral infiltrates, respiratory insufficiency, or ARDS. Alveolar hemorrhage has been described in a few cases, including one report in a patient with HIV and severe ehrlichiosis, where post-mortem evaluation revealed alveolar hemorrhage and vascular congestion [7]. Alveolar hemorrhage can result from the systemic inflammatory response to infection. While rare in ehrlichiosis, this phenomenon has been associated with infections such as influenza, leptospirosis, dengue, and malaria in immunocompetent individuals [8]. Additionally, while kidney injury can be seen with ehrlichiosis, in this case, it was suspected to be multifactorial, with exposure to nephrotoxic agents and hypotension from shock as primary factors. Ehrlichia infections can result in kidney injury by causing reduced blood flow from decreased blood pressure, direct infection of nephrons, and immune complex deposition in glomeruli secondary to systemic inflammatory response [7]. Advanced age and delay in therapy are known risk factors for severe disease, as demonstrated in this case.

Diagnosis of ehrlichiosis can be a challenge due to nonspecific clinical and laboratory manifestations, incomplete exposure histories, limited awareness of endemic regions, and reduced access to infectious disease specialists [9]. Tick-borne illnesses are a growing health concern as tick populations are growing and distributions are expanding, driven in part by climate change [10]. Clinicians must maintain a high index of suspicion for tick-borne disease in endemic areas, particularly during peak seasons or with a history of animal or outdoor exposures. Early recognition, timely diagnostic confirmation, and prompt initiation of doxycycline therapy are necessary to decrease the risk of severe disease and complications.

## Conclusions

This case is noteworthy because it demonstrates the importance of detailed history-taking, the value of pattern recognition, and the critical role infectious disease specialists play in guiding patient care. Additionally, it highlights a rare but serious complication of ehrlichiosis, alveolar hemorrhage, in an immunocompetent patient. As demonstrated in this case, increased awareness of ehrlichiosis and its potential complications can result in improved patient outcomes through timely diagnosis and treatment.
